# Analysis of extracellular vesicle mRNA derived from plasma using the nCounter platform

**DOI:** 10.1038/s41598-021-83132-0

**Published:** 2021-02-12

**Authors:** Jillian W. P. Bracht, Ana Gimenez-Capitan, Chung-Ying Huang, Nicolas Potie, Carlos Pedraz-Valdunciel, Sarah Warren, Rafael Rosell, Miguel A. Molina-Vila

**Affiliations:** 1 Pangaea Oncology, Laboratory of Oncology, Quirón Dexeus University Hospital, Sabino Arana 5-19, 08028 Barcelona, Spain; 2 Department of Biochemistry, Molecular Biology and Biomedicine, Universitat Autónoma de Barcelona (UAB), 08193 Cerdanyola, Spain; 3 NanoString Technologies, Seattle, WA USA; 4 Department of Genetics, Faculty of Science, University of Granada, 18071 Granada, Spain; 5 Bioinformatics Laboratory, Biotechnology Institute, Centro de Investigacion Biomedica, PTS, Avda. del Conocimiento s/n, 18100 Granada, Spain; 6 Germans Trias i Pujol Health Sciences Institute and Hospital (IGTP), Badalona, Barcelona Spain

**Keywords:** Gene expression analysis, Tumour biomarkers, Biomarkers, Cancer, RNA

## Abstract

Extracellular vesicles (EVs) are double-layered phospholipid membrane vesicles that are released by most cells and can mediate intercellular communication through their RNA cargo. In this study, we tested if the NanoString nCounter platform can be used for the analysis of EV-mRNA. We developed and optimized a methodology for EV enrichment, EV-RNA extraction and nCounter analysis. Then, we demonstrated the validity of our workflow by analyzing EV-RNA profiles from the plasma of 19 cancer patients and 10 controls and developing a gene signature to differentiate cancer versus control samples. TRI reagent outperformed automated RNA extraction and, although lower plasma input is feasible, 500 μL provided highest total counts and number of transcripts detected. A 10-cycle pre-amplification followed by DNase treatment yielded reproducible mRNA target detection. However, appropriate probe design to prevent genomic DNA binding is preferred. A gene signature, created using a bioinformatic algorithm, was able to distinguish between control and cancer EV-mRNA profiles with an area under the ROC curve of 0.99. Hence, the nCounter platform can be used to detect mRNA targets and develop gene signatures from plasma-derived EVs.

## Introduction

With the growing global cancer burden, estimated at 18 million cases in 2018^1^, earlier cancer detection, enhanced disease monitoring and improved therapy selection are indispensable to enhance patient survival. Liquid biopsies provide a minimally invasive, safe and sensitive surrogate for tissue biopsies. Extracellular vesicles (EVs) are double-layered phospholipid membrane vesicles that are released by most cells, including cancer cells, immune cells and even blood platelets, and can be isolated from practically any body fluid. Cells liberate highly heterogeneous EVs in terms of size (10 nm–1 μm), cargo (nucleic acids, proteins and lipids), membrane composition, biogenesis and biological function^2–4^. Several EV enrichment strategies have been described, including ultracentrifugation, size-exclusion chromatography and precipitation. Precipitation buffers capture water molecules and thereby decrease the hydration of particles, allowing their precipitation after a low-speed centrifugation.

Importantly, the active molecules that are found within EVs can be transported to local or distant target cells and execute biological functions, making EVs important mediators of intercellular communication^2,5,6^. Since the quantity of released EVs and their specific cargo is regulated by the producing cells, the RNA profiles contained within EVs could potentially be used as biomarkers for development and progression of several diseases, including cancer^7^. EVs also have the advantage of their lipid bilayer, which makes their cargo particularly stable and allows the use of biobank stored samples.

Gene expression studies using EV-RNA from cancer patients are an active area of research^8,9^. Promising findings have been reported but the lack of standardized protocols for RNA extraction and analysis, leading to inconsistent results, is hampering clinical implementation. Transcriptomic analysis studies are often conducted using quantitative real time PCR (qRT-PCR), microarrays or RNA sequencing (RNAseq), each with their own (dis)advantages^10^. While qRT-PCR provides high sensitivity and specificity with a short turnaround time, it only allows for low-throughput transcriptomic analysis of a limited number of genes. Microarrays represent a medium-throughput platform but have a narrower dynamic range of detection and are not suitable to detect genes expressed at either low or high levels. Finally, RNAseq is an accurate, high-throughput platform with a wide dynamic range, but limitations include longer turnaround time, high cost and complex data analysis^11,12^.

In recent years, the NanoString nCounter platform has gained popularity in translational research and clinical settings. The platform provides a simple, sensitive and cost-effective solution for multiplexed analysis of up to 800 RNA targets by direct capturing and counting of individual targets. In addition, it can be used with formalin-fixed paraffin embedded (FFPE) tumor tissue and allows for low quality and quantity tissue samples^13,14^. At this respect, the nCounter-based Prosigna assay, which differentiates breast cancer subtypes and predicts the risk of recurrence based on a 50-gene signature, has been validated in the clinical practice and received FDA approval in 2013^15,16^. Another assay developed using the nCounter platform is the 18-gene Tumor Inflammation Signature (TIS), which was able to predict clinical response to PD-1 blockade in an investigational clinical trial assay^17^. These two assays, which utilize tissue samples, emphasize the potential of the nCounter platform as biomarker assay development tool, especially in diagnostic laboratories. Regarding the analysis of liquid biopsies on nCounter, several studies have investigated the potential of some materials, including cf-^18^ and EV-DNA^19^, CTC-RNA^20,21^, leukocyte mRNA^22^, cfRNA^23^ and EV-miRNA^24,25^, with different success rates. However, nCounter has never been tested for the analysis of EV-derived mRNA.

Here, we present a proof-of-concept study where we optimized a workflow for EV enrichment from human blood samples, EV-mRNA purification and subsequent analysis by nCounter. Then, we used the workflow to develop an EV-mRNA based gene signature to differentiate cancer versus control samples. Our work demonstrates that nCounter can be employed for biomarker discovery based on EV-mRNA.

## Results

### Optimization of plasma EV enrichment and EV-RNA extraction methodologies

**Figure 1:**
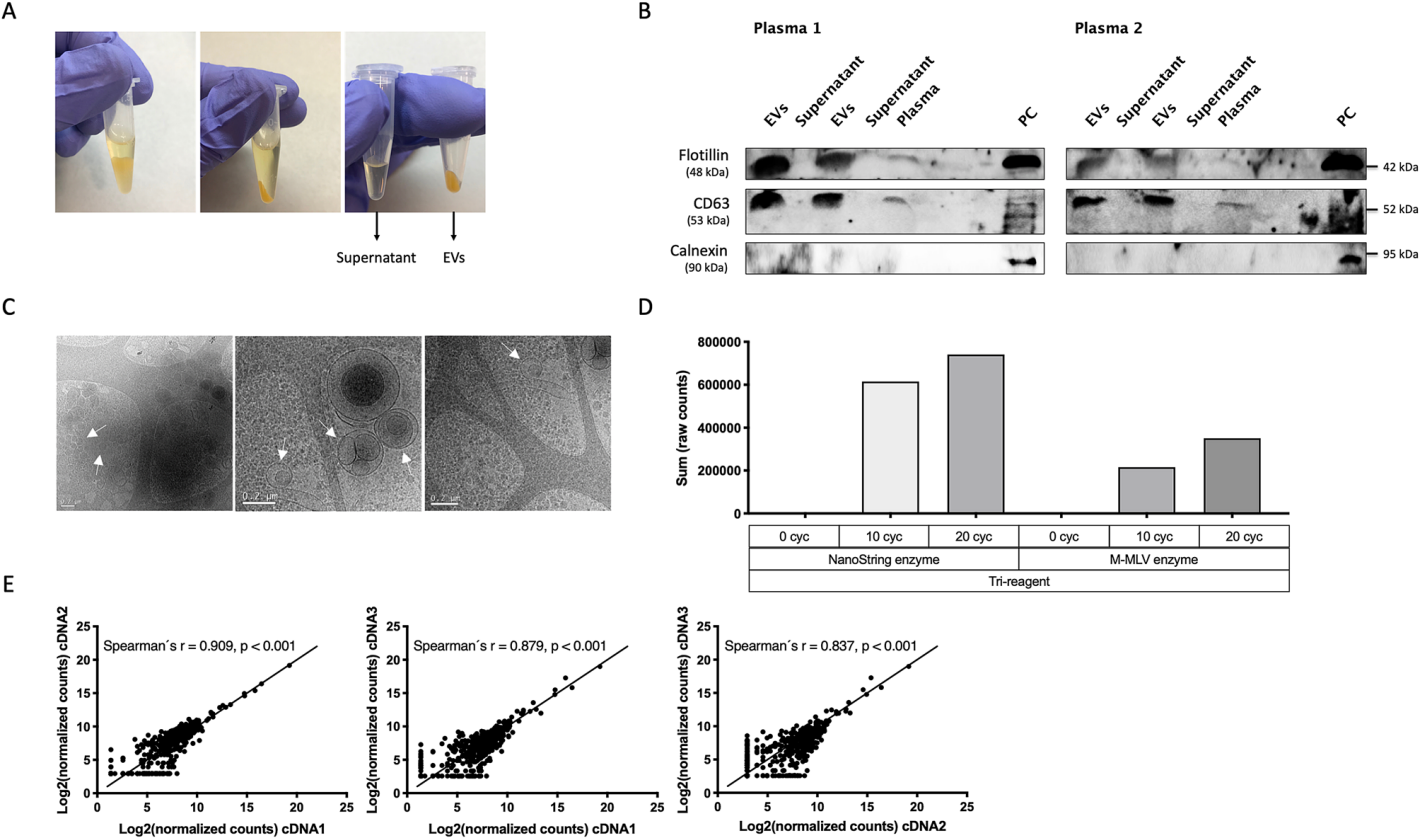
EV enrichment and characterization, assay reproducibility. (**A**) EV enrichment based on precipitation using the miRCURY Exosome Serum/Plasma Kit, including separation of supernatant and EV-enriched pellet. (**B**) Immunoblots showing expression of Flotillin, CD63 (both exosome markers) and Calnexin (cell specific marker) in EV-enriched pellets, supernatants, full plasma and a positive control sample. Experiments were performed in duplicates. Membranes were cut and incubated with specific antibodies for Flotillin, CD63 and Calnexin. Images were cropped for clarity purposes and full membranes can be found in Supplementary Fig. S1. (**C**) Cryogenic Electron Microscopy (cryo-EM) of EV pellets. Arrows point to extracellular vesicles with different size ranges. Scale bars are 200 nm. (**D**) Total counts by nCounter after EV-RNA extraction using TRI reagent. Two different retrotranscriptases (NanoString versus M-MLV) and three pre-amplification conditions (0, 10 and 20 cycles) were tested. (**E**) Reproducibility experiment comparing the Log2 normalized counts by nCounter from three independent cDNAs derived from a single EV-RNA sample. Spearman’s correlation coefficient is indicated. EVs, extracellular vesicles; PC, positive control; Cyc, cycles.

EVs were enriched from 500 µL plasma of control samples using the miRCURY Exosome Serum/Plasma Kit (Fig. 1a). Final miRCURY sediments were submitted to western blotting, revealing enrichment in the exosome markers Flotillin and CD63, which were absent or detected at low levels in miRCURY supernatants and whole plasma samples. Sediments, supernatants and plasmas were negative for the cell-specific marker calnexin (Fig. 1b, Supplementary Fig. S1). Cryogenic electron microscopy (cryo-EM), a commonly used technique for EV characterization^26,27^, was used to visualize the miRCURY sediments, revealing EVs with the classical morphology and a diameter of 100–300 nm, in agreement with the reported 10–1 µm size range (Fig. 1c)^2–4^.

TRIzol LS and TRI reagent are mixtures of phenol, guanidine isothiocyanate and other components routinely used for nucleic acid extractions. We found that the quantity of RNA that could be isolated from EV-enriched sediments using TRI reagent was too low to be determined by the Qubit RNA High Sensitivity Assay Kit (Thermo Fisher Scientific). Bioanalyzer profiles of two representative samples revealed RNA concentrations < 150 pg/μL, insufficient for nCounter analysis using the Human Immunology V2 Panel, which we had selected for our study (Supplementary Fig. S2). Therefore, we tested retrotranscription and pre-amplification of the EV-mRNA with the nCounter Low RNA Input Amplification Kit, using primers targeting the genes of the Panel. Two reverse transcriptases were compared, the M-MLV and the enzyme provided by the kit, together with 10 versus 20-cycles for the pre-amplification step. Results indicated that the retrotranscriptase provided by the kit was more efficient in terms of final counts and that both 10 and 20 cycles yielded sufficient raw counts for successful nCounter analysis with the Human Immunology V2 Panel (Fig. 1d). However, 20-cycles of pre-amplification led to saturation for some genes with higher expression levels (Supplementary Fig. S2). In consequence, 10 cycles were selected for the final workflow.

To test the reproducibility of the steps described above, we retrotranscribed and pre-amplified the same EV-RNA sample on three independent reactions. Then, we compared the results obtained when submitting the three resulting cDNA samples to nCounter analysis. A strong correlation was found between the normalized counts for each individual gene obtained in the different cDNAs, represented by a Spearman’s r of 0.84–0.91, *p* < *0.01* (Fig. 1e).

**Figure 2:**
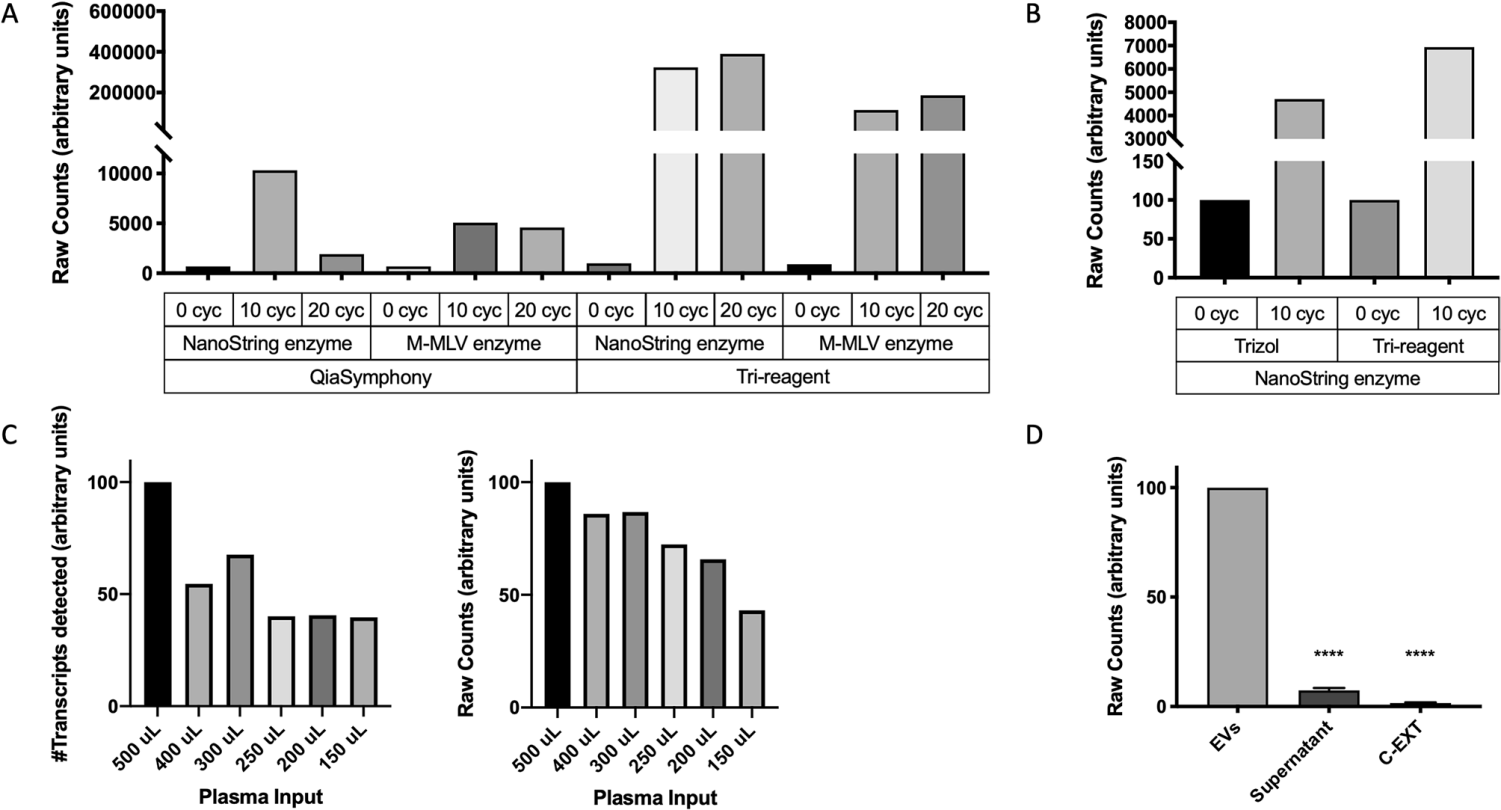
EV-RNA extraction, targeted pre-amplification and plasma input testing. (**A**) Total nCounter counts after automated (QiaSymphony) versus manual (TRI reagent) RNA extraction from an EV-enriched pellet. Two different retrotranscriptases (NanoString versus M-MLV) and three pre-amplification conditions (0, 10 and 20 cycles) were tested. Results were normalized to the counts corresponding to 0 cycles. (**B**) Total nCounter counts after TRIzol LS versus TRI reagent manual RNA isolation from an EV-enriched pellet. Results were normalized to the counts corresponding to 0 cycles. (**C**) Effect of input plasma volume (150–500 μL) on the final number of transcripts detected (left) or total nCounter counts (right) Results were normalized to the counts corresponding to 500 μL plasma. (**D**) Total nCounter counts of different fractions obtained during EV enrichment of plasma (EVs vs. supernatant *p* < *0.0001* in a one-way ANOVA with Dunnett’s multiple comparisons test; EVs versus C-EXT *p* < *0.0001*). Cyc, cycles; EVs, extracellular vesicles; C-EXT, extraction control.

All the experiments so far described had been performed with 500 μL plasma samples and TRI reagent. Two additional RNA extraction methods were tested on EV-enriched preparations from control samples, the automated QiaSymphony and the manual TRIzol LS Reagent based isolation (Fig. 2a). When considering the total number of counts by nCounter, TRI reagent was found to outperform both QiaSymphony and TRIzol LS, independently of the retrotranscriptase or the number of cycles used for the pre-amplification step (Fig. 2b). Finally, we also tested the effect of plasma input volume on downstream analysis of EV-RNA on the nCounter platform. Both the total counts and number of transcripts detected were higher with an initial plasma volume of 500 μL (Fig. 2c).

RNase A is a bovine enzyme that can be used to degrade RNA in a sample. When enriching for EVs from plasma samples, we expect two sources of RNA; extra-vesicular RNA and RNA derived from within the EVs. Our workflow for EV-RNA analysis incorporates an RNase A treatment of the EV-enriched pellets in order to remove extra-vesicular RNA not embedded into the particles. To further validate this step, we collected two EV-enriched pellets and the corresponding supernatants of healthy patients, treated them with RNase A, purified the RNA and analyzed them with the Human Immunology V2 Panel. We found that EV-enriched pellets yielded significantly higher transcript counts compared to the supernatant (one-way ANOVA with Dunnett’s multiple comparisons, EVs versus supernatant *p* < *0.01*), indicating that the transcripts detected by nCounter are associated with EVs (Fig. 2d). To validate the efficacy of our RNase treatment, we also checked the expression of *GAPDH* and *CCL5* by qRT-PCR in three patient samples. Three different sample conditions were used; EVs without RNase treatment, EVs with RNase treatment and lysed EVs with RNase treatment. Results indicated that RNase treatment of intact EVs slightly reduced the *GAPDH* and *CCL5* mRNA levels, probably by removing the extra-vesicular RNA co-precipitated with the EVs (Supplementary Fig. S3). In contrast, once EVs were lysed, RNase treatment effectively eliminated all RNA and both *GAPDH* and *CCL5* transcripts were undetectable, confirming that transcripts purified using our workflow are indeed contained within the EVs.

### EV-derived mRNA analysis on nCounter and classifier development

**Figure 3:**
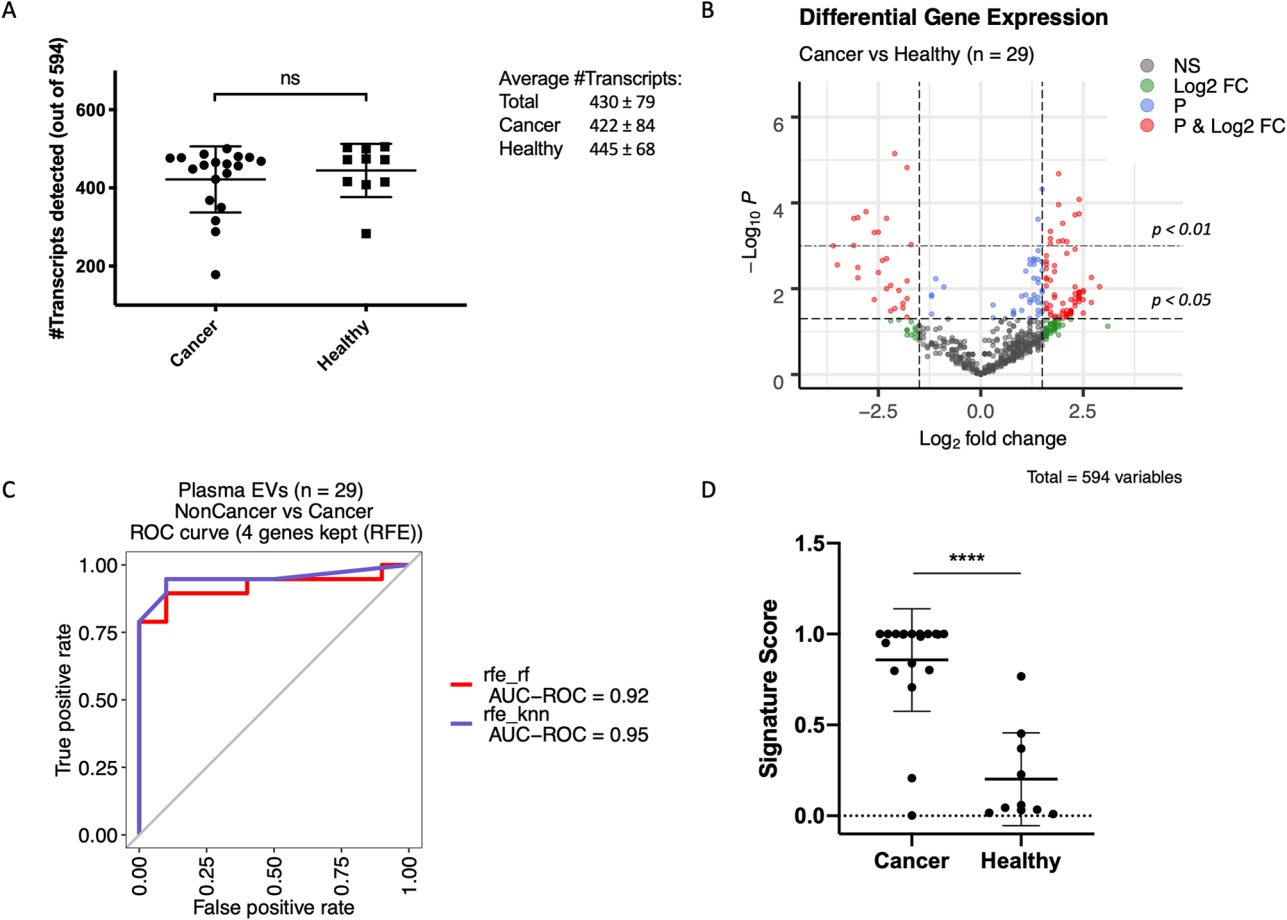
EV-mRNA transcript detection, differential expression analysis and development of a classifier algorithm. (**A**) Number of transcripts detected in EVs from cancer patients and healthy controls using the Human Immunology v2 nCounter panel, which targets 594 genes (two-tailed Mann–Whitney U test, *p* = *0.463*). (**B**) Differential expression analysis of log2-normalized counts between cancer patients and healthy controls. The full list of genes differentially expressed is presented in Supplementary Table S1. (**C**) Area under the ROC curve of the four-gene signature, selected using recursive feature elimination (RFE), to differentiate cancer from control samples. (**D**) Scores of cancer versus control samples based on expression of the four-gene signature (*p* < *0.001* in a two-tailed Mann–Whitney U test). NS, not significant; EVs, extracellular vesicles; ROC, receiver operating characteristic; AUC, area under the curve; RFE, recursive feature elimination; rf, random forest; knn, k-nearest neighbors.

Based on the data presented above, we selected a workflow for subsequent experiments; starting with EV enrichment from 500 μL of plasma with the miRCURY kit, followed by manual RNA purification with TRI reagent, retrotranscription with the Nanostring enzyme, a 10-cycle pre-amplification and final cDNA analysis by nCounter. We validated the proposed workflow by studying the EV-mRNA expression of 19 cancer patient and 10 control plasma samples using the Human Immunology V2 Panel (Table 1). If all samples were considered together, the average number of transcripts detected was 430 ± 79 out of the 594 transcripts in the panel (Fig. 3a). No significant differences were found between the number of mRNA transcripts in EVs from controls versus cancer patients (445 ± 68 and 422 ± 84 respectively, Mann–Whitney’s U *p* = *0.46*).

**Table 1:** Clinical characteristics of the cancer patients and controls included in the study.

Characteristics	Cancer patients (n = 19)	Controls (n = 10)
**Gender—no. (%)**		
Male	11 (57.9)	5 (50.0)
Female	8 (42.1)	5 (50.0)
**Age—year**		
Median	62	42
Range	45–78	24–53
**Tumor type—no. (%)**		
Lung (ADC)	10 (52.7)	–
Gastric (ADC)	3 (15.8)	–
Anal (SCC)	2 (10.5)	–
Rectal (ADC)	2 (10.5)	–
Sarcoma (UPS)	2 (10.5)	–
**Stage—no. (%)**		–
Stage III	2 (10.5)	–
Stage IV	17 (89.5)	–

ADC, adenocarcinoma; SCC, squamous cell carcinoma; UPS, undifferentiated pleomorphic sarcoma.

After normalization, we analyzed the differential expression (DE) of transcripts in EVs from cancer patients versus controls (Fig. 3b, Supplementary Table S1). We found 141 mRNAs with significantly different levels; of them, 107 were upregulated and 34 downregulated in the EVs from cancer patients versus controls. Then, we used a recursive feature elimination (RFE) method to select a gene signature predictive of the origin of the sample; a cancer patient or a control sample. The transcripts included in the final signature were *BCL10*, *CXCL11*, *CYBB* and *GBP1* and, based on their expression levels, our algorithm was able to classify plasma samples into cancer and control with a receiver operating characteristic (ROC) area under the curve (AUC) of 0.92 to 0.95 (Fig. 3c). The classifier also calculated signature scores for each sample, which were found to be significantly different between cancer patients and controls (Mann–Whitney U, *p* < *0.01*; Fig. 3d).

### Improvement of classifier performance through removal of genomic DNA

**Figure 4:**
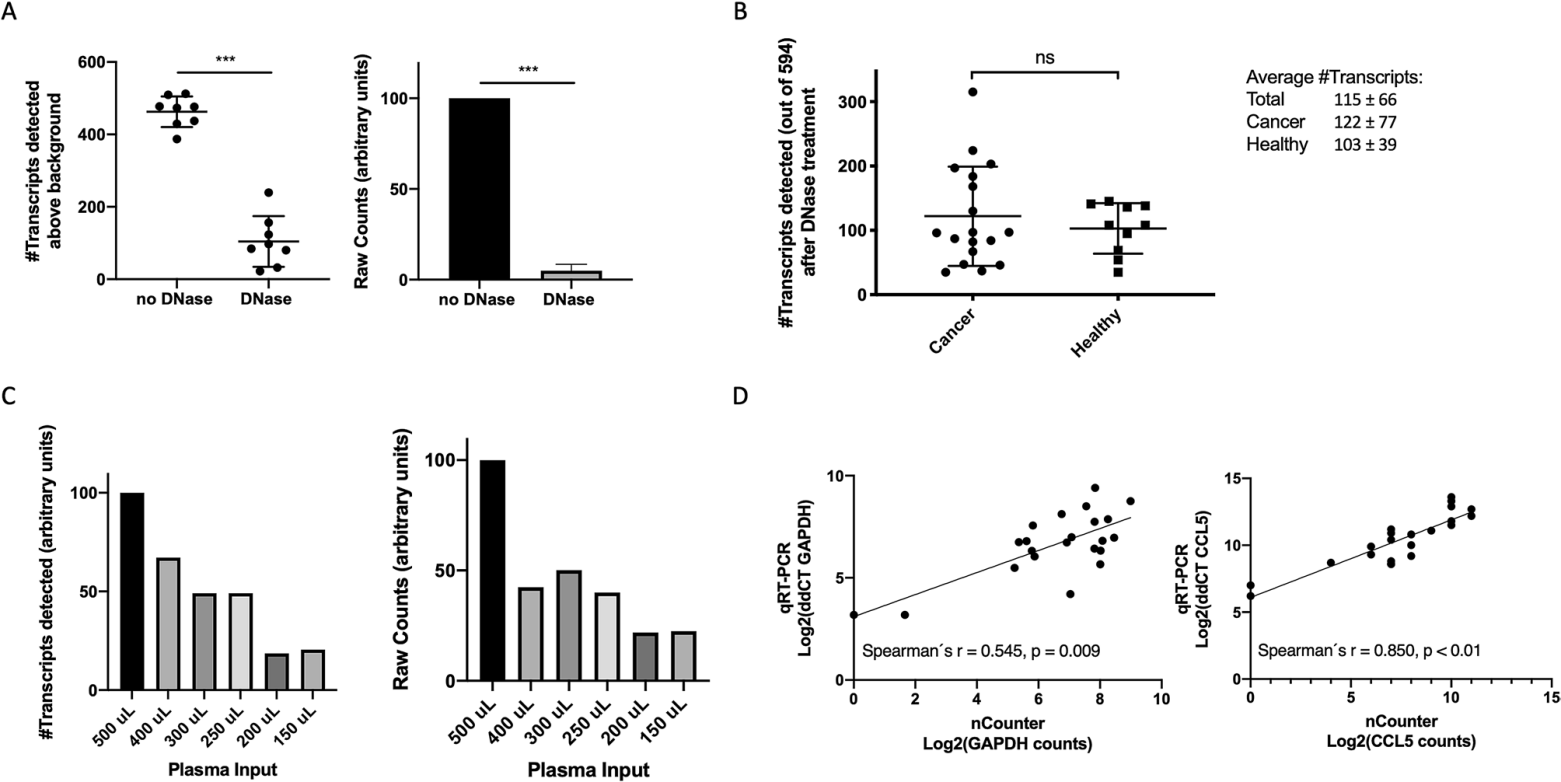
Effect of DNase treatment on the number of transcripts detected, total counts, and additional validation experiments. (**A**) Number of transcripts detected (left) and total nCounter counts (right) in non-DNase vs. DNase treated EV samples (*p* < *0.001* in a two-tailed Mann–Whitney U test, in both cases). (**B**) Number of transcripts detected in EVs from cancer patients and control samples using the Human Immunology v2 nCounter panel, after DNase treatment (*p* = *0.916* in a two-tailed Mann–Whitney U test). (**C**) Effect of input plasma volume (150–500 μL) on the final number of transcripts detected (left) or total nCounter counts (right), after DNase treatment. Results were normalized to the counts corresponding to 500 μL plasma. (**D**) Comparison of Log2 counts by nCounter versus ddCT values by qRT-PCR for *GAPDH* and *CCL5* in 22 samples. Spearman’s correlation coefficient is indicated. NS, not significant.

The TRI reagent-based RNA isolation incorporated in our workflow for EV-RNA purification may also co-extract EV-genomic DNA, which could bind to the nCounter probes during hybridization. In consequence, some of the counts detected can correspond to genomic DNA instead of mRNA transcripts, particularly considering that we used a 10-cycle pre-amplification step. To test if this was the case, we analyzed the effect of adding a DNase treatment step after EV-RNA extraction in the 28 eV samples with remaining material. A dramatic reduction in the number of transcripts detected and the total amount of counts was observed in the EV-RNA samples after DNase treatment (Fig. 4a, Mann–Whitney U, *p* < *0.01* in both cases). The average number of transcripts in the 28 samples decreased from 430 ± 79 to 115 ± 66 and the total counts dropped more than 90%. As previously observed in samples without DNase treatment, the number of transcripts detected in cancer versus control samples were not significantly different (122 ± 77 vs 103 ± 39, Mann–Whitney’s U *p* = *0.92*, Fig. 4b). In addition, there were no significant differences in the number of transcripts detected between the different tumor types; which included sarcoma, lung, rectal, anal and gastric cancer (Supplementary Fig. S4).

**Figure 5:**
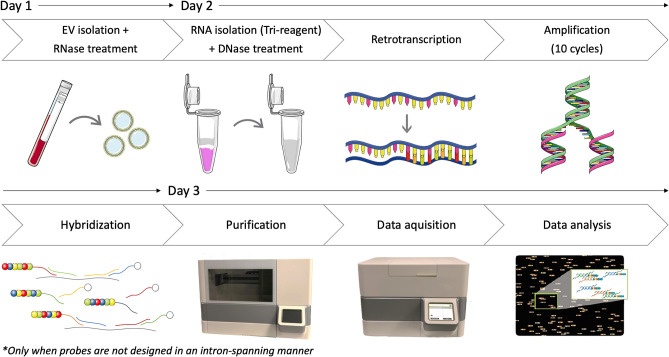
Final workflow for EV-RNA extraction and analysis on the nCounter platform. The miRCURY kit was used to enrich EVs from 500 μL plasma and EV-enriched preparations were treated with RNase to remove extra-vesicular RNA. Then, EV-RNA was extracted using TRI reagent and treated with DNase to remove genomic DNA. DNase treatment is only necessary when probes are not designed in an intron-spanning manner. Next, we performed retrotranscription and a 10-cycle pre-amplification, followed by hybridization, purification on the nCounter prep-station and analysis on the nCounter digital analyzer. EVs, extracellular vesicles. Part of this figure was modified from SMART (Servier Medical Art), licensed under a Creative Common Attribution 3.0 Generic License. http://smart.servier.com/.

Based on these results, we decided to incorporate a DNase step in our EV-mRNA purification and analysis workflow when working with this panel (Fig. 5), and we confirmed that 500 μL still yielded the highest number of transcripts and total counts (Fig. 4c). To further validate the DNase step, we analyzed *GAPDH*, *CCL5* and *Caspase 8 (CASP8)* expression by qRT-PCR in the 22 eV samples with remaining RNA. We observed a statistically significant correlation between *GAPDH* and *CCL5* expression as measured by nCounter and qRT-PCR (Spearman’s r = 0.545, *p* < *0.01* and r = 0.850, *p* < *0.01*, respectively; Fig. 4d); while *CASP8* transcripts were undetectable in DNase treated EV-RNA by both techniques.

**Figure 6:**
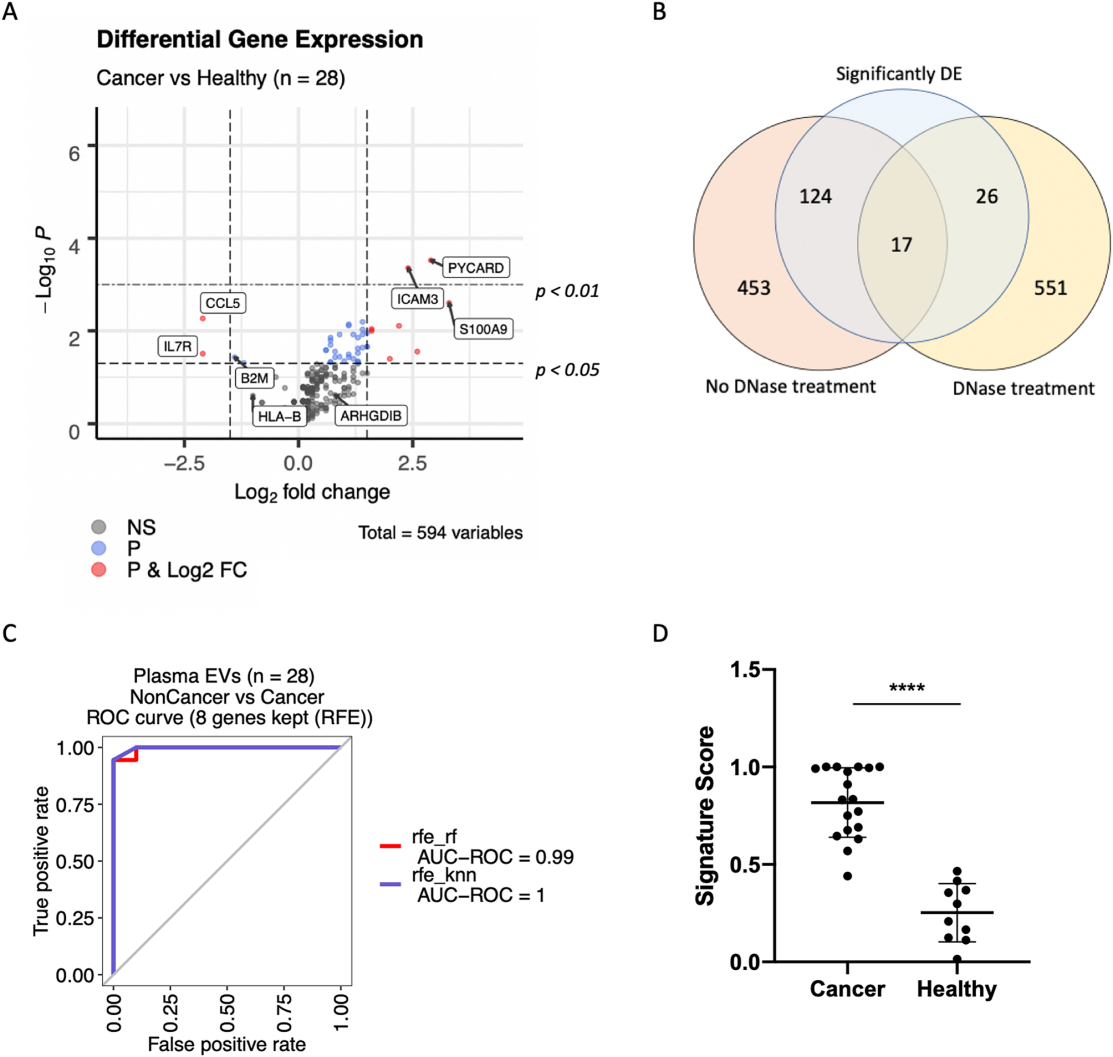
Effect of DNase treatment on differential expression analysis and classifier development. (**A**) Differential expression analysis of log2-normalized counts after DNase treatment between cancer patients and control samples. The full list of genes differentially expressed is presented in Supplementary Table S2. Labels indicate the eight transcripts selected for the final classification signature. (**B**) Out of 594 genes in the panel, 17 showed differential expression independently of DNase treatment. (**C**) Recursive feature elimination (RFE) was used to select an eight-gene signature that could distinguish between samples derived from cancer patients and controls. (**D**) Scores of cancer versus control samples based on expression of the eight gene signature (p < 0.001 in a two-tailed Mann–Whitney U test). DE, differentially expressed; EVs, extracellular vesicles; ROC, receiver operating characteristic; AUC, area under the curve; RFE, recursive feature elimination; rf, random forest; knn, k-nearest neighbors.

Next, we proceeded to redesign our gene signature and classifier algorithm using the expression data derived from DNase treated EV-mRNAs. We observed that the number of differentially expressed genes in cancer versus control samples dropped from 141 to 43 (Fig. 6a and Supplementary Table S2). Of them, 17 genes (40%) overlapped with those obtained using non-DNase treated samples (Fig. 6b). Then, we used the RFE algorithm to create a second signature, which included eight genes: *CCL5, S100A9, B2M, HLA-B, IL7R, ICAM3, ARHGDIB and PYCARD* (Fig. 6a and Supplementary Table S3). The algorithm based on this signature was able to classify the samples into cancer and control with a ROC AUC of 0.99 to 1.00 (Fig. 6c). Also, the signature scores for each sample were found to be significantly different between cancer patients and controls (Mann–Whitney U, *p* < *0.01*; Fig. 6d).

The significant decrease in counts after DNase treatment prompted us to further investigate the binding of nCounter probes to genomic DNA co-purified with EV-mRNA. The nCounter Human Immunology V2 Panel targets 594 gene transcripts. The probes for some genes are designed within the same exon (“non-intron spanning”); while other probes (“intron spanning”) target sequences corresponding to contiguous exons of the cDNA/mRNA. Genomic DNA should only bind “non-intron spanning” probes, and we performed an additional experiment to confirm this point. We analyzed an EV sample in quadruplicate, skipping the retrotranscriptase and/or the DNase treatment (Supplementary Table S4). Without DNase treatment, we observed counts for “non-intron spanning” probes (such as *CASP8*, *TNFRSF8* and *B2M*) both in presence and in absence of the retrotranscriptase step. In contrast, counts for “intron spanning” probes (such as *PRKCD*, *TNFRSF10C*, *FCER1G*) were apparent only if retrotranscription was performed. Finally, DNase treatment induced a significant drop in the counts of “non-intron spanning” but, unexpectedly, also “intron spanning” probes.

## Discussion

The molecules found within EVs, such as mRNAs, are often involved in intercellular communication and represent a potential source for biomarker discovery. However, lack of standardized methods and clinical validation prevents the implementation of EV-derived testing in daily practice. The nCounter platform, which allows for multiplex detection of hundreds of transcripts, has been extensively used in translational research for transcriptomic tumor characterization. In addition, the nCounter Prosigna assay, based on a 50-gene expression signature, has been fully standardized and validated at the clinical level; and received FDA approval in 2013 to predict risk of recurrence in breast cancer^15,16,28^. However, studies investigating the performance of nCounter for mRNA analysis in liquid biopsies are scarce, particularly in the case of EVs.

Here, we present a workflow for nCounter-based analysis of plasma-derived EVs and we demonstrate that it can be used to efficiently detect transcripts in EV-enriched preparations from cancer patients and control samples. The first step of the workflow is plasma processing using the precipitation-based miRCURY kit which, as previously described^29,30^, yielded pellets enriched in EVs. Clinical laboratories do not usually have access to ultracentrifugation and precipitation kits offer several advantages, such as short turn-around time and limited technical requirements. Although 500 μL plasma was found to give the highest amount of detected transcripts and total counts by nCounter, lower plasma inputs also yielded valid results. The second step is manual RNA isolation using TRI reagent, which was found to outperform manual purification by TRIzol LS and automated extraction.

The extraction methods tested, including the TRI reagent based manual extraction, did not yield sufficient RNA for direct analysis by nCounter. In consequence, after some optimization experiments, a 10-cycle pre-amplification step was added to our workflow and found to be highly reproducible. Most RNA extraction methods are known to co-purify genomic DNA (gDNA), and this was also our case. The EV-associated gDNA could be located in the interior of the vesicles or be attached to the membranous surface, and further research is needed to clarify this issue. More importantly, it has been described that simultaneous isolation of EV-derived genomic DNA during EV-RNA extraction can affect the amount of detected transcripts^31^. In consequence, we tested the effect of adding a DNase treatment step to our methodology and found a significant decrease in total counts and number of transcripts detected. Interestingly, while the expression of many genes became undetectable after DNase treatment, the counts for some transcripts were maintained. These observations suggested that co-extracted EV-genomic DNA was amplified during the pre-amplification step and could hybridize to the “non-intron spanning” probes in the nCounter panel. In contrast, probes designed in an “intron-spanning” manner should not hybridize to EV-DNA. Validation experiments confirmed this point (Supplementary Table S4). Unexpectedly, we observed a sharp decrease also in the counts of “intron-spanning” probes after DNase treatment, strongly suggestive of EV-RNA degradation or, more likely, EV-RNA loss during the purification steps needed to remove the DNase. In consequence, appropriate design of probes should be preferred over DNase treatment when analyzing samples with low amounts of RNA that require pre-amplification. Since we were using a pre-designed panel that could not be modified, we added a DNase treatment step to our final protocol for EV-mRNA analysis (Fig. 5). As an additional validation of the entire workflow, we re-analyzed 22 DNase treated samples for *GAPDH, CASP8* and *CCL5* expression by qRT-PCR. Similarly to previous reports^12,32^, we found a statistically significant correlation between the expression levels obtained by qRT-PCR and nCounter.

Next, we investigated if our workflow for EV-mRNA analysis could be used to develop gene signatures. To this end, we performed differential expression analysis of the nCounter results obtained for control and cancer samples. The signature-based algorithm obtained for EV-mRNA (DNase treated) showed an improved classifier performance in comparison with EV-nucleic acids (non-DNase treated); with ROC-AUCs of 0.99–1.00 versus 0.92–0.95 for the discrimination of control versus cancer, respectively. This result is coincident with a previous report where a DNA removal step was shown to reduce signature noise during algorithm development and yielded better classification results^33^. The genes selected for our EV-mRNA expression signature are *CCL5*, *S100A9*, *B2M*, *HLA-B*, *IL7R*, *ICAM3*, *ARHGDIB* and *PYCARD*; which are involved in several immune-related pathways such as cytokine signaling, innate immune system or lymphocyte activation (Fig. 6a and Supplementary Table S3). *CCL5*, *B2M*, *HLA-B* and *IL7R* are all related to cytokine signaling and lymphocyte activation, and are thus known as immunomodulators. Interestingly, these four transcripts were downregulated in cancer samples, suggesting differences in immune system activation through cytokine induction between cancer patients and controls. *PYCARD* is also proposed to be involved in lymphocyte activation and was found to be upregulated in cancer patients. A previous study found that *PYCARD* could suppress apoptosis of cancer cells in gastric cancer^34^, potentially explaining the observed higher expression in EVs from cancer patients.

Two studies have used nCounter for analysis of mRNA isolated from blood. Kossenkov et al^23^ developed a pulmonary node classifier to differentiate malignant from non-malignant nodules previously detected by low-dose CT. Since the authors made use of whole blood, the quantities of purified RNA were significantly higher than those in our EV-based study (3 μg/2.5 mL blood versus 0.01 μg/2.5 mL, respectively), avoiding the need for a pre-amplification step. Beck et al^21^ used a combination of CTC-RNA and cfRNA to profile tumor-associated biomarkers and correlate them with diagnostics and survival. The quantities of RNA were similar to those obtained in our study and a pre-amplification step was also added.

Our study shows several limitations. First, although purification of EVs from plasma yielded pellets that were found to be enriched in EVs, such precipitation techniques can also isolate a significant fraction of proteins and lipoproteins^35,36^. Second, there are no validated housekeeping genes for normalization of EV-mRNAs and we had to use the total amount of counts, as described^37–39^. Third, although nCounter presents many advantages, it has also a few limitations when compared to other multiplex techniques such as RNAseq. For instance, since nCounter works with gene panels, no new transcripts can be found. Finally, our aim was to establish a workflow for the nCounter analysis of EV-mRNA and the mixed patient population we used to demonstrate the validity of our approach was not the most appropriate to develop a clinically useful signature. Much larger patient cohorts would be needed to train and validate signatures that could differentiate between cancer patients and controls.

In summary, to the best of our knowledge, this proof-of-concept study is the first to demonstrate that the nCounter platform can be used to reproducibly detect plasma EV-mRNA transcripts. Differential expression analysis can then be implemented for biomarker assay development. Our work paves the way for widespread testing of EV-mRNA expression in blood and other fluids, and subsequent selection of signatures useful in the clinical setting.

## Methods

### Patient samples

This study was carried out in accordance with the principles of the Declaration of Helsinki under an approved protocol of the institutional review board of Quirón Hospitals. We selected for the study all blood samples from advanced stage cancer patients arriving to our institution in a two month period with sufficient volume for EV extraction after routine genetic testing (n = 19). Blood samples from 10 healthy controls were also collected (Table 1). Written informed consent was obtained from all participants and documented; samples were de-identified for confidentiality. Clinical information collected from each participant was limited to gender, age, tumor type and stage.

### Extracellular vesicle enrichment

Whole blood samples (10 mL) were collected in sterile EDTA Vacutainer tubes (BD, Plymouth, UK) and centrifugated twice at 1000 × g for 10 min at room temperature (RT). Plasma samples were stored at − 80 °C until further processing. The miRCURY Exosome Serum/Plasma Kit (Qiagen, Hilden, Germany) was used to enrich for EVs from 500 μL plasma, according to the manufacturer’s instructions, unless otherwise specified. In short, dead cells and debris (including platelets and fibrin) were cleared with thrombin and centrifugation. Precipitation Buffer was added, samples were incubated overnight at 4 °C and the EV fraction was pelleted by centrifugation. Supernatants were collected and stored for separate analysis. EV enriched pellets were resuspended for further processing.

### EV characterization by western blot

EV enriched pellets were resuspended in 300 μL ice-cold radioimmunoprecipitation assay buffer containing protease inhibitor mixture (Roche Applied Science, Penzberg, Germany), as previously described^40^. Samples were incubated on ice for 30 min, homogenized and centrifugated 15 min, 12.000 × g at 4 °C. Supernatants (lysates) were collected and 80 μg proteins were electrophoresed on 10% SDS–polyacrylamide gels (Life Technologies, Carlsbad, CA, USA) and transferred to PVDF membranes (Bio-Rad Laboratories Inc., Hercules, CA, USA). Membranes were blocked in Odyssey Blocking Buffer (Li-Cor Biosciences, Lincoln, NE, USA). All target proteins were immunoblotted with appropriate primary and horseradish peroxidase (HRP)-conjugated secondary antibodies (Supplementary Table S5). Chemiluminescent bands were detected in a ChemiDoc MP Imaging System (Bio-Rad Laboratories Inc.).

### EV characterization with cryogenic electron microscopy

Cryogenic Electron Microscopy (Cryo-EM) was performed by the Microscopy Service of the Universitat Autonoma de Barcelona (UAB), and used for direct visualization of EVs using the TEM JEOL 2011 200 kV. As previously described^41^, 2 µL volume of the resuspended EV sample was added to a carbon TEM grid. The grid was transferred onto the cyro-preparation chamber of a Leica electron microscope, containing a liquid ethane bath cooled to -180 °C. Using a piece of filter paper, the EV solution was taken off the grid and plunged into the liquid ethane. The orifice trapped frozen EV solution was assembled into a plunger (Leica EM GP) and blotted with Whatman No. 1 filter paper. The grid was placed in a liquid nitrogen bath, and then loaded into a liquid nitrogen-cooled TEM grid holder. The grid holder was placed into a JEOL 2011 TEM microscope. Imaging was performed using a Gatan UltraScan US1000 CCD camera and data was analyzed with Digital Micrograph 1.8.

### RNA extraction

EV-enriched pellets were treated with 4 μg/mL RNase A from bovine pancreas (Sigma-Aldrich, St. Louis, MO) for 1 h at 37 °C, to remove extra-vesicular RNA not associated to EVs. For TRIzol LS Reagent (Thermo Fisher Scientific, Waltham, MA) and TRI Reagent (MRC, Cincinnati, OH) extraction, TRIzol solutions were added to a final volume of 1 mL and incubated at RT for 20 min to inactivate RNase A and lyse the EVs. Then, 200 μL Chloroform: Isoamyl Alcohol (24:1) (Panreac Química SLU, Barcelona, Spain) was added and samples were vigorously vortexed and centrifuged at 12,000 × g for 15 min at 4 °C. The aqueous upper layer was kept and RNA was precipitated adding 2.5 μL Glycogen (Merck KGaA, Darmstadt, Germany) and 500 μL 2-propanol (Merck KGaA), incubating 10 min at RT and centrifugating for 10 min, 12,000 × g, at 4 °C. The final RNA pellet was washed with 75% ethanol, air dried and dissolved in 20 μL nuclease free water. The QIAsymphony DSP Virus/Pathogen Kit was also tested in the automated QIAsymphony SP System (Qiagen) for RNA extraction from EVs, according to the manufacturer’s instructions.

### Electrophoretic analysis of RNA

The approximate quantity and size distribution of the isolated EV-RNA was evaluated using the Bioanalyzer RNA 6000 Pico Assay (Agilent Technologies, Santa Clara, CA) according to manufacturer instructions.

### DNase treatment

In order to remove co-isolated DNA, the EV-RNA samples were treated with the DNA-free DNA Removal Kit (Thermo Fisher Scientific), according to manufacturer instructions. In short, 1 μL DNase buffer and 0.5 μL enzyme were added to 7.5 μL RNA sample, followed by incubation at 37 °C for 30 min and DNase removal.

### Gene expression analysis using nCounter

The nCounter Low RNA Input Amplification Kit (NanoString Technologies, Seattle, WA) was used to retrotranscribe and pre-amplify 4 μL EV-derived RNA using 10 cycles. Retrotranscription was carried out in 0.5 mL tubes while pre-amplification, using primers targeting the genes of the Human Immunology V2 Panel (NanoString Technologies), was performed in 384-well plates to prevent sample evaporation. In parallel, a Moloney Murine Leukema Virus (M-MLV) Reverse Transcriptase Enzyme (Thermo Fisher Scientific) was also tested for cDNA synthesis. The Human Immunology V2 Panel (NanoString Technologies) was used to analyze EV-derived, pre-amplified cDNA according to manufacturer instructions. This panel targets 594 general genes involved in the immune response such as cytokines, enzymes, interferons and their receptors. Samples were hybridized for 18 h at 65 °C.

### Data normalization and analysis

Raw nCounter counts of expressed genes were normalized in R and R studio (version 3.6.3, https://cran.r-project.org/bin/macosx/) using the R package *NanoStringNorm*^42^. Normalization was performed following several steps: technical assay variability normalization using the geometric mean of the positive control probes, background correction using the mean plus two times standard deviation (SD) of the negative control probes, and sample content normalization using the total amount of counts for each sample. Normalized counts were log2-transformed, and used for differential expression (DE) analysis. Log2 fold change (FC) of each gene was calculated as the ratio of average log-2 transformed counts of the cancer patient cohort versus the control cohort. Volcano plots were used to visualize log2 FC on the x-axis and nominal p-values on the y-axis. GraphPad Prism software (version 9.0.0; https://www.graphpad.com/scientific-software/prism/) was used for other statistical testing and to create figures.

### Classifier algorithm development

Optimal gene selection was performed using recursive feature elimination (RFE). To this end, a leave-one-out cross validation (LOOCV) algorithm was used on the full Human Immunology V2 gene Panel. The number of genes to select was set at 4, 8, 16 or 579 and the amount of genes that yielded optimal performance after cross-validation was automatically selected. Classification was performed with the selected gene signature using random forest (RF) and k-nearest neighbors (KNN) classifiers with three iterations. The model with the highest accuracy was then selected as the final model. Signature scores for each sample were derived from the final model.

### Gene expression analysis using qRT-PCR

Complementary DNA (cDNA) was synthesized using the M-MLV Reverse Transcriptase Enzyme (Thermo Fisher Scientific). Hereafter, cDNA was added to TaqMan Universal Master Mix (Applied Biosystems) in a 12.5 μL reaction with specific primers and probe designed for each gene. The primer and probe sets were designed using Primer Express Software (version 3.0.1; https://www.thermofisher.com/order/catalog/product/4363993#/4363993) according to their Ref Seq (http://www.ncbi.nlm.nih.gov/LocusLink) Gene-specific primers were designed as follows: GAPDH, forward: 5′-TGACCTCAACTACATGGTTTACATGTT-3′ and reverse: 5′-TGACGGTGCCATGGAATTT-3′; Caspase 8, forward: 5′-CAGGGCTCAAATTTCTGCCTAC-3′ and reverse: 5′-GAAGAAGTGAGCAGATCAGAATTGAG-3′; CCL5, forward: 5′ CATCTGCCTCCCCATATTCCT 3′ and reverse: 5′ AGTGGGCGGGCAATGTAG 3′. Quantification of gene expression was performed using the QuantStudio 7 Flex Real-Time PCR System (Thermo Fisher Scientific). Expression levels of mRNA were expressed as arbitrary units based on Ct values. Commercial RNAs were used as controls (liver and lung; Stratagene, La Jolla, CA). In all quantitative experiments, a sample was considered not evaluable when the standard deviation of the Ct values was > 0.30 in 2 independent analyses.

## Supplementary information

Supplementary Information.

## Data Availability

Supplementary information is available for this paper.
