# Supplementary material for: Analysis of extracellular vesicle mRNA derived from plasma using the nCounter platform
Source: Sci Rep. 2021 Feb 12;11:3712. doi: 10.1038/s41598-021-83132-0 (PMC7881020; doi:10.1038/s41598-021-83132-0)
Supplement: Supplementary file 1 — Supplementary Information. [file 41598_2021_83132_MOESM1_ESM.docx]

**EXTENDED DATA**

**Analysis of extracellular vesicle mRNA derived from plasma using the nCounter platform.**

Jillian WP Bracht^1,2*^, Ana Gimenez-Capitan^1^, Chung-Ying Huang^3^, Nicolas Potie^4,5^, Carlos Pedraz-Valdunciel^2,6^, Sarah Warren^3^, Rafael Rosell^6^, Miguel A Molina-Vila^1*^

**FIGURES**

*
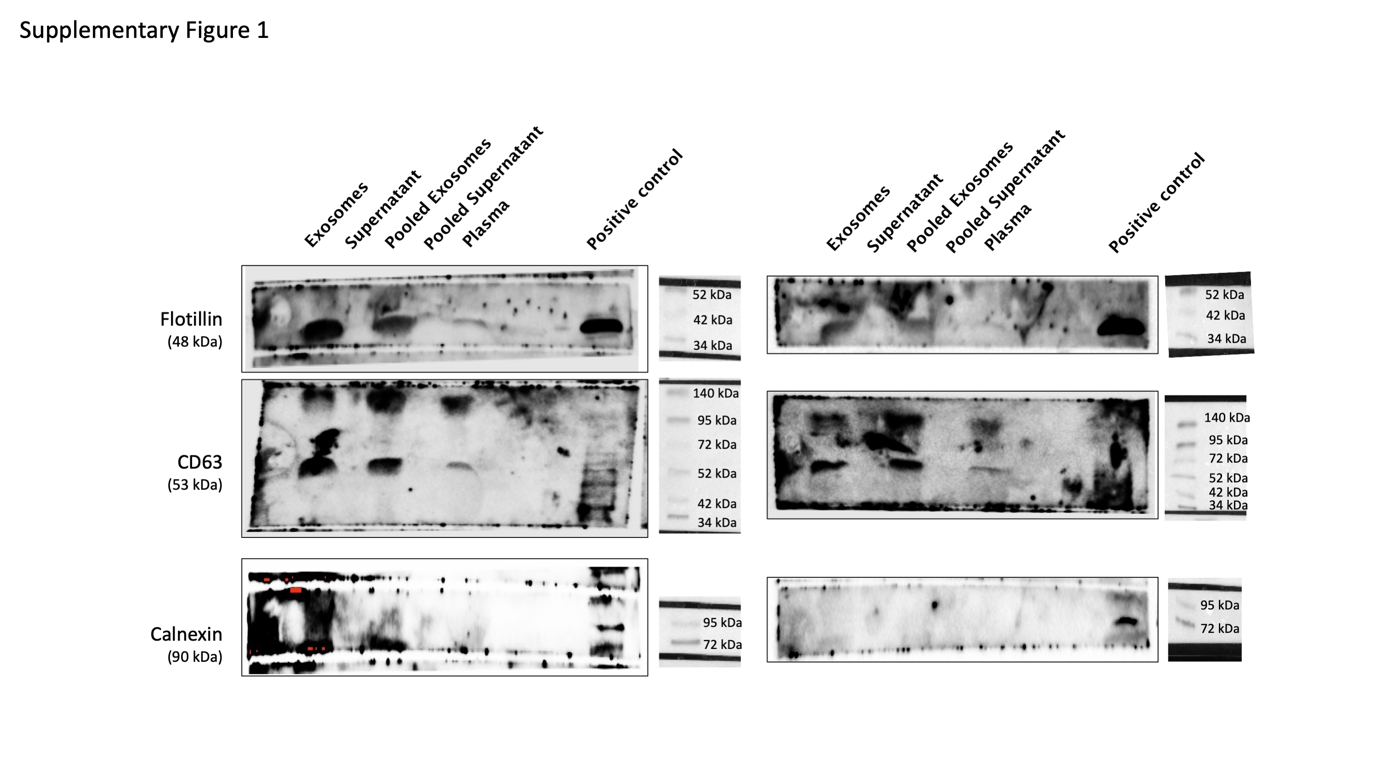
*

**Supplementary Figure S1. Full immunoblot membranes showing expression of Flotillin, CD63 and Calnexin in EV-enriched pellets, supernatants, full plasma and a positive control sample.**

**
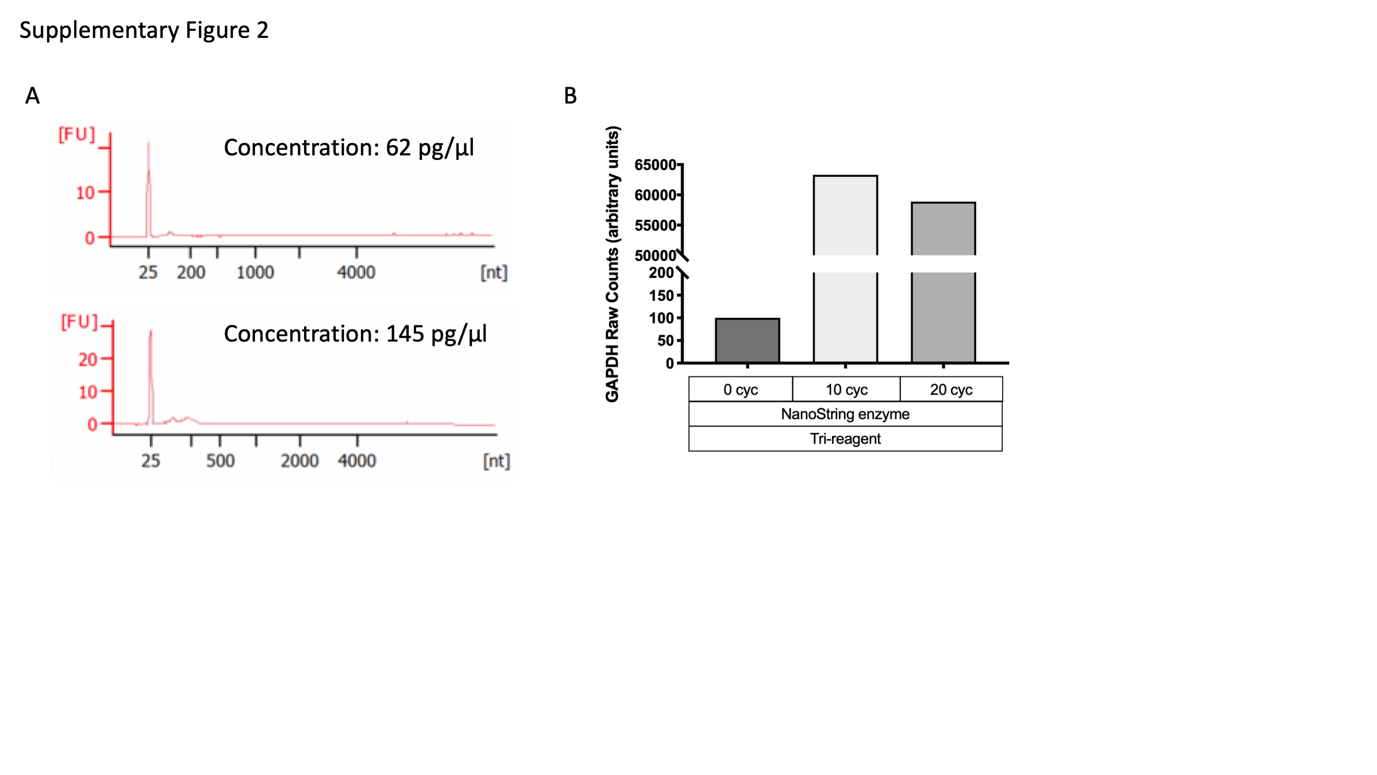
**

**Supplementary Figure S2. Bioanalyzer profiles for RNA quantity and quality and saturation of GAPDH transcripts after pre-amplification. (A)** Bioanalyzer profiles of Tri-reagent based EV-RNA extraction from 500 μL plasma. **(B)** Raw GAPDH counts after Tri-reagent based EV-RNA extraction. Different pre-amplification conditions (0, 10 and 20 cycles) were tested, using the same plasma sample. The 0 cycle condition was used as control value for normalization. A pre-amplification of 20 cycles was found to cause saturation in the GAPDH counts. *Cyc: cycles.*

**
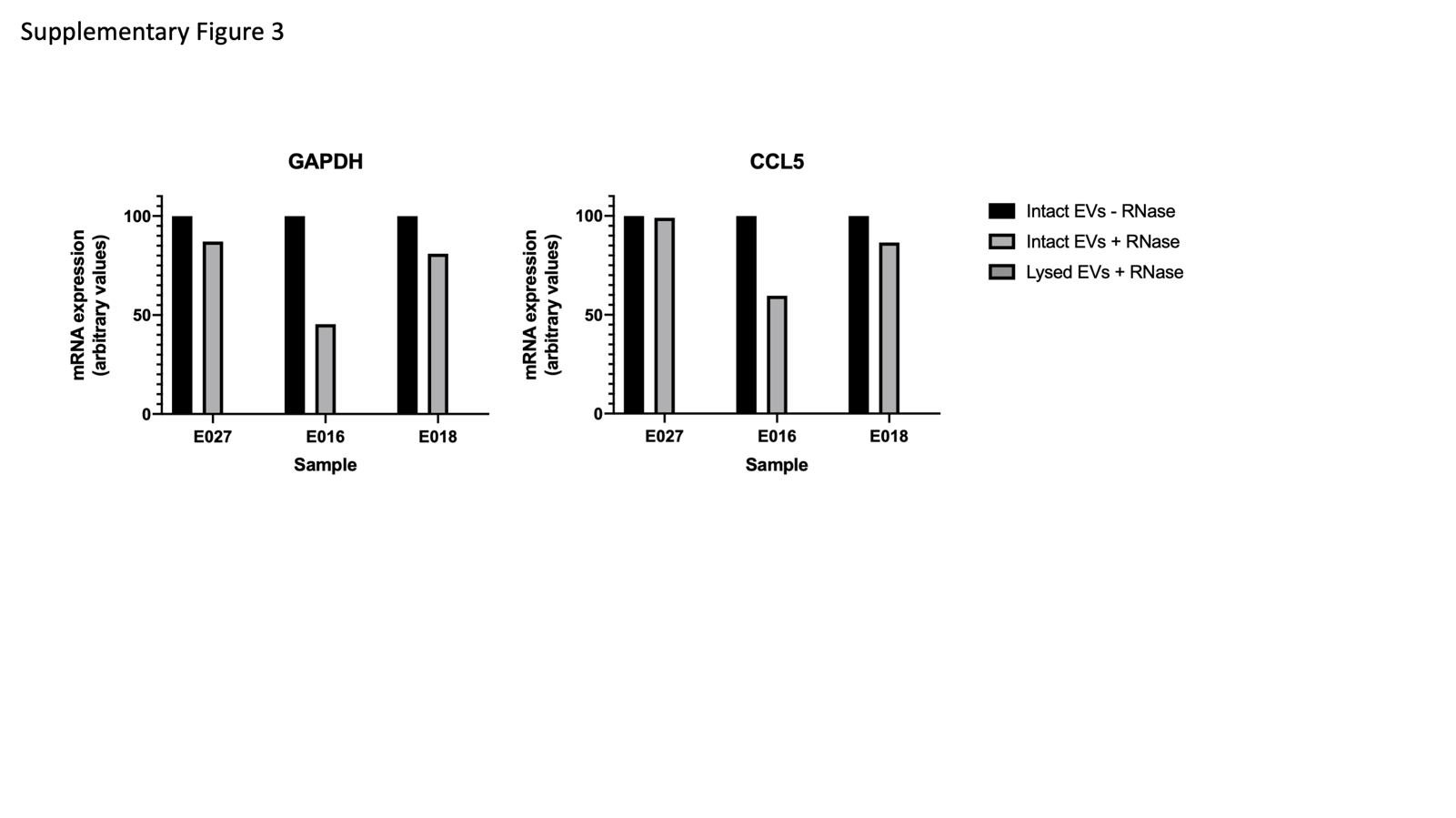
**

**Supplementary Figure S3. qRT-PCR analysis for *GAPDH* and *CCL5* in three patient samples to determine the efficacy of RNase A treatment.** For each patient three different sample conditions were used: intact EVs with- and without RNase treatment, and lysed EVs after RNase treatment. Results were normalized to the counts corresponding to EVs without RNase treatment. Undetermined values by qRT-PCR have been assigned a value of 0%.

**
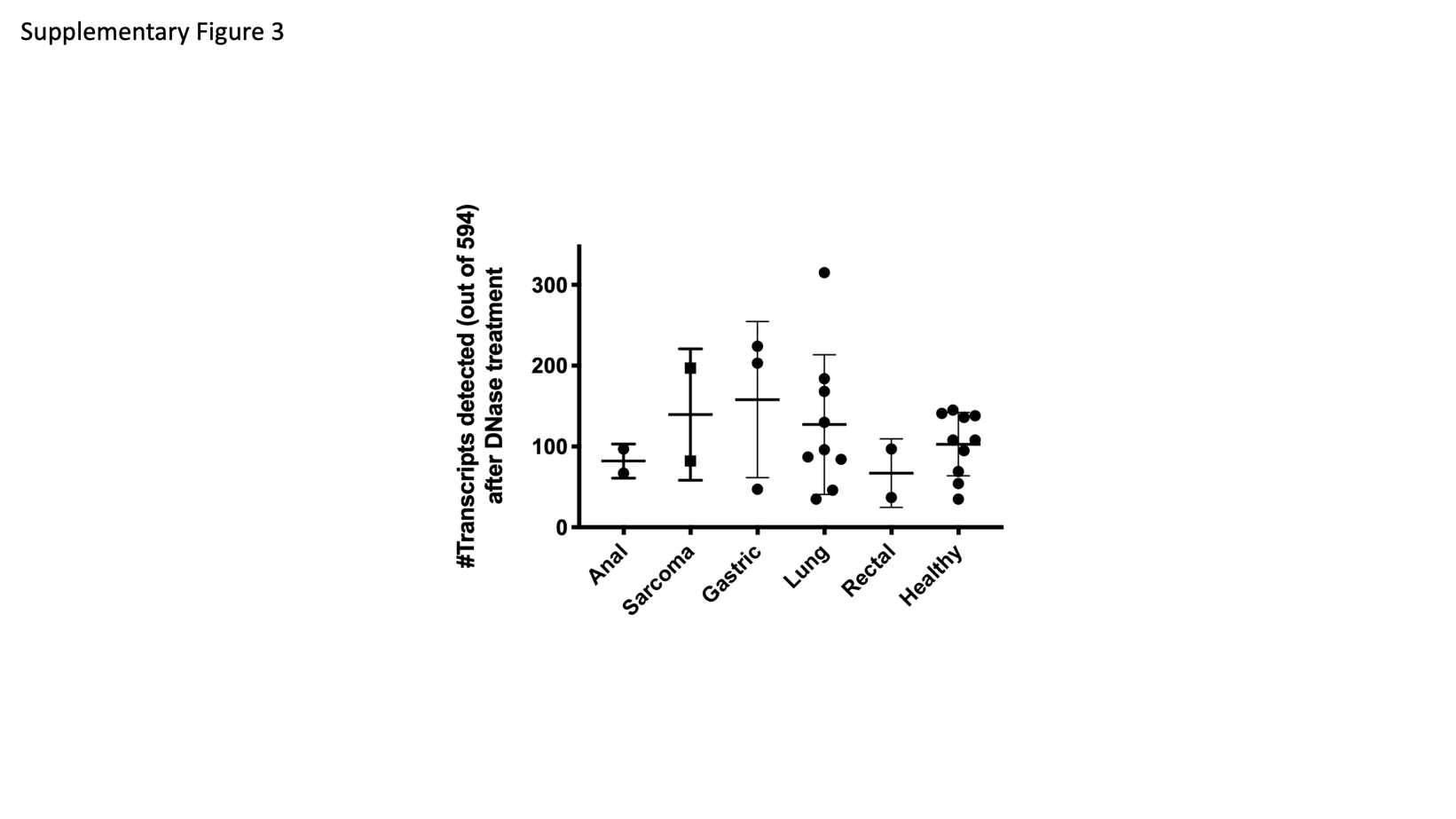
**

**Supplementary Figure S4. Number of transcripts detected in EVs from cancer patients, divided by tumor type, and control samples using the Human Immunology v2 nCounter panel, after DNase treatment (p = 0.756 in a Kruskal-Wallis test).**

**TABLES**

**Supplementary Table S1.** Transcripts differentially expressed in EVs of cancer patients vs. controls (*p < 0.05*). A log Fold Change (FC) > 0 corresponds to enriched mRNAs in EVs of cancer patients.

*LogFC: logarithmic Fold Change.*

| **Target** | **LogFC** | **Pvalue** |
| --- | --- | --- |
| LEF1 | -4,30 | 3,10E-06 |
| B2M | -2,10 | 7,10E-06 |
| CCL5 | -1,80 | 1,50E-05 |
| HLA-DRB3 | 1,90 | 2,10E-05 |
| IL18RAP | 4,60 | 3,70E-05 |
| CYBB | 1,50 | 4,80E-05 |
| BCL10 | 2,40 | 8,30E-05 |
| TNFSF13B | 1,90 | 0,00011 |
| MIF | -2,80 | 0,00016 |
| IFNG | 2,40 | 0,00018 |
| AHR | 2,30 | 0,00019 |
| ITGAE | -3,00 | 0,00022 |
| PTK2 | -2,30 | 0,00023 |
| BCAP31 | -3,10 | 0,00023 |
| STAT1 | 1,40 | 0,00024 |
| IL28A | 2,00 | 3,00E-04 |
| CD59 | 1,70 | 0,00046 |
| APP | -2,50 | 0,00048 |
| CD44 | -2,60 | 0,00049 |
| PRF1 | 1,70 | 0,00068 |
| CXCL11 | 2,00 | 0,00076 |
| CCRL1 | 1,90 | 0,00079 |
| GBP1 | 2,10 | 8,00E-04 |
| KIR3DL2 | 1,70 | 0,00089 |
| PPBP | -1,70 | 0,00094 |
| IL4R | -3,10 | 0,00098 |
| HLA-B | -3,60 | 0,001 |
| MME | 2,30 | 0,0012 |
| ADA | 1,40 | 0,0013 |
| BTLA | 2,10 | 0,0015 |
| CD36 | 1,60 | 0,0017 |
| IL7R | 1,30 | 0,002 |
| PECAM1 | -2,30 | 0,002 |
| CASP8 | 1,40 | 0,0021 |
| SELL | 1,20 | 0,0021 |
| GPI | 1,30 | 0,0022 |
| CD99 | -2,40 | 0,0022 |
| SMAD3 | 1,60 | 0,0023 |
| TIRAP | 1,50 | 0,0024 |
| IRAK4 | 1,60 | 0,0027 |
| IRF7 | 1,30 | 0,0027 |
| TAL1 | 1,20 | 0,0027 |
| CD27 | -3,50 | 0,0028 |
| IL28A/B | 1,80 | 0,0029 |
| CD9 | -3,00 | 0,0032 |
| SPP1 | 1,60 | 0,0034 |
| CCR6 | 1,50 | 0,0038 |
| TAGAP | 1,10 | 0,0039 |
| TRAF6 | 1,80 | 0,004 |
| C1QBP | -2,50 | 0,0042 |
| LILRA6 | 2,70 | 0,0055 |
| CHUK | 1,60 | 0,0056 |
| CD79A | -3,00 | 0,0056 |
| MYD88 | 1,30 | 0,0057 |
| C4A/B | 1,40 | 0,0059 |
| FCER1G | -1,10 | 0,0059 |
| IFNA1/13 | 1,60 | 0,006 |
| TRAF1 | 1,70 | 0,0064 |
| HLA-DMA | -1,80 | 0,0066 |
| FCGR1A/B | 1,40 | 0,0072 |
| PSMB10 | -2,20 | 0,0084 |
| DEFB4A | 1,60 | 0,0086 |
| HFE | 2,90 | 0,009 |
| CXCL1 | 2,30 | 0,0091 |
| OAZ1 | -0,90 | 0,0091 |
| CASP3 | -2,30 | 0,01 |
| CCL15 | 2,50 | 0,011 |
| ICOSLG | 1,50 | 0,011 |
| CD3D | -2,00 | 0,011 |
| CLEC7A | 2,50 | 0,012 |
| CXCR4 | 2,40 | 0,012 |
| IL13RA1 | 2,40 | 0,012 |
| IL20 | 1,50 | 0,012 |
| C6 | 2,40 | 0,013 |
| SOCS3 | 2,30 | 0,013 |
| CCL13 | 1,80 | 0,014 |
| ATG5 | 1,30 | 0,014 |
| GZMA | 1,10 | 0,014 |
| HLA-A | -1,20 | 0,014 |
| FADD | 2,40 | 0,015 |
| GZMB | 1,00 | 0,015 |
| KLRB1 | -1,20 | 0,015 |
| CCRL2 | 2,40 | 0,016 |
| IL12RB1 | 1,80 | 0,016 |
| PDGFB | 1,40 | 0,016 |
| XCL1 | 2,40 | 0,017 |
| CFI | 2,30 | 0,017 |
| CTSC | -1,80 | 0,017 |
| IL2 | 2,50 | 0,018 |
| C8B | 2,20 | 0,018 |
| ITGAL | 1,30 | 0,018 |
| ETS1 | 1,20 | 0,018 |
| CXCL12 | -2,60 | 0,018 |
| ARG2 | 2,40 | 0,02 |
| PTAFR | 2,70 | 0,021 |
| SLAMF1 | 2,30 | 0,021 |
| BID | 1,40 | 0,021 |
| IL1RN | 1,20 | 0,021 |
| IL15 | 2,00 | 0,022 |
| PPIA | 1,60 | 0,022 |
| CSF1 | 1,50 | 0,022 |
| LY96 | -1,90 | 0,022 |
| SIGIRR | 0,30 | 0,024 |
| CTLA4_all | 2,30 | 0,025 |
| ATG10 | 1,70 | 0,025 |
| CD7 | -1,90 | 0,028 |
| IFNB1 | 1,60 | 0,029 |
| IL26 | 1,60 | 0,029 |
| IL2RG | -2,10 | 0,029 |
| CCR8 | 2,20 | 0,032 |
| LTA | 2,10 | 0,032 |
| MR1 | 1,40 | 0,032 |
| PSMB7 | 1,00 | 0,032 |
| MBL2 | 2,20 | 0,033 |
| GATA3 | 2,00 | 0,033 |
| C14orf166 | 0,80 | 0,033 |
| TFRC | -2,20 | 0,033 |
| NOD2 | 2,20 | 0,034 |
| TNFRSF13C | 1,50 | 0,034 |
| LILRB4 | 1,70 | 0,035 |
| CXCR2 | 2,20 | 0,036 |
| MASP1 | 2,50 | 0,037 |
| CTNNB1 | 0,80 | 0,037 |
| IFNA2 | 2,20 | 0,038 |
| IL1A | 2,20 | 0,038 |
| SELE | 2,00 | 0,038 |
| C9 | 2,10 | 0,039 |
| FCER1A | -1,20 | 0,039 |
| CD19 | 2,10 | 0,04 |
| NFATC3 | 0,80 | 0,04 |
| CCL18 | 1,70 | 0,041 |
| CXCR6 | 1,40 | 0,041 |
| LILRA4 | 2,10 | 0,044 |
| IL8 | 1,90 | 0,044 |
| LILRB2 | 1,80 | 0,044 |
| VCAM1 | 2,10 | 0,045 |
| IKZF2 | -1,80 | 0,046 |
| MBP | 1,80 | 0,047 |
| MAPK1 | 0,30 | 0,048 |
| LAMP3 | 2,00 | 0,049 |
| CD83 | 1,20 | 0,049 |

**Supplementary Table S2.** Transcripts differentially expressed in EVs of cancer patients vs. controls (*p < 0.05*) after DNase treatment. A log Fold Change (FC) > 0 corresponds to enriched mRNAs in EVs of cancer patients.

*LogFC: logarithmic Fold Change.*

| **Target** | **LogFC** | **Pvalue** |
| --- | --- | --- |
| PYCARD | 2,90 | 3,00E-04 |
| ICAM3 | 2,40 | 0,00044 |
| S100A9 | 3,30 | 0,0025 |
| PRKCD | 2,10 | 0,0025 |
| CCL5 | -2,10 | 0,0054 |
| IL2RG | 1,40 | 0,0064 |
| IRF7 | 1,10 | 0,0072 |
| FCGR2A | 1,10 | 0,0077 |
| PSMB10 | 2,20 | 0,0078 |
| FKBP5 | 1,60 | 0,0091 |
| ARG1 | 1,40 | 0,0093 |
| GPI | 1,60 | 0,01 |
| ADA | 1,50 | 0,011 |
| LITAF | 1,20 | 0,012 |
| HLA-C | 1,40 | 0,012 |
| TUBB | 0,90 | 0,012 |
| C1QBP | 0,70 | 0,014 |
| RPL19 | 1,30 | 0,014 |
| KLRF1 | 0,70 | 0,016 |
| CD7 | 0,70 | 0,02 |
| S100A8 | 1,50 | 0,022 |
| MIF | 1,50 | 0,022 |
| CD59 | 1,40 | 0,023 |
| STAT1 | 1,30 | 0,024 |
| BATF3 | 0,60 | 0,026 |
| GBP5 | 0,60 | 0,026 |
| HLA-DPB1 | 2,60 | 0,028 |
| TAPBP | 0,80 | 0,028 |
| PTPN6 | 1,30 | 0,03 |
| IL7R | -2,10 | 0,031 |
| NCF4 | 1,10 | 0,032 |
| TBK1 | 0,90 | 0,036 |
| LTF | 1,10 | 0,037 |
| B2M | -1,40 | 0,037 |
| XBP1 | 0,80 | 0,037 |
| BCL10 | 1,20 | 0,04 |
| GAPDH | 2,00 | 0,04 |
| HLA-DMA | 1,30 | 0,045 |
| IL8 | 1,00 | 0,045 |
| CTSG | 0,70 | 0,047 |
| IFNGR1 | 0,70 | 0,047 |
| C1QB | 1,30 | 0,047 |
| PTK2 | -1,20 | 0,049 |

**Supplementary Table S3.** Transcripts included in the eight-gene signature. A log Fold Change (FC) > 0 corresponds to enriched mRNAs in EVs of cancer patients. Annotations for each transcript, as provided by the panel manufacturer, are also presented.

*LogFC: logarithmic Fold Change.*

| **Target** | **LogFC** | **Pvalue** | **Annotation** | | | | | | | | | | | | | | | | | |
| --- | --- | --- | --- | --- | --- | --- | --- | --- | --- | --- | --- | --- | --- | --- | --- | --- | --- | --- | --- | --- |
|  |  |  | Adaptive Immune System | Apoptosis | Cell Adhesion | Chemokine Signaling | Cytokine Signaling | Host-pathogen Interaction | Inflammasomes | Innate Immune System | Lymphocyte Activation | Lymphocyte Trafficking | MHC Class I Antigen Presentation | NLR signaling | Oxidative Stress | Phagocytosis and Degradation | TNF Family Signaling | TLR Signaling | Type I Interferon Signaling | Type II Interferon Signaling |
| ARHGDIB | 0.8 | 0.24 | - | + | - | - | - | - | - | - | - | + | - | - | - | - | - | - | - | - |
| B2M | -1.4 | 0.04 | + | - | - | - | + | - | - | + | + | - | + | - | - | - | - | - | - | + |
| CCL5 | -2.1 | < 0.01 | - | - | - | + | + | + | - | - | + | - | - | + | + | - | + | + | - | - |
| HLA-B | -1.0 | 0.25 | + | - | + | - | + | + | - | + | + | - | + | - | - | + | - | - | + | + |
| ICAM3 | 2.4 | < 0.01 | + | - | + | - | - | - | - | + | - | - | - | - | - | - | - | - | - | - |
| IL7R | -2.1 | 0.03 | - | - | - | - | + | - | - | - | + | - | - | - | - | - | - | - | - | - |
| PYCARD | 2.9 | < 0.01 | - | - | - | - | - | + | + | + | + | - | - | + | - | - | - | - | - | - |
| S100A9 | 3.3 | < 0.01 | - | - | - | - | - | - | - | + | - | - | - | - | - | - | - | + | - | - |

**Supplementary Table S4.** Effect of probe design on binding of EV-cargo. Upper panel, treatments tested. Lower panel, nCounter counts obtained for 6 transcripts, three recognized by non-intron-spanning (CASP8, TNFRSF8 and B2M) and three by intron-spanning probes (PRKCD, TNFRSF10C and FCER1G).

*EVs: extracellular vesicles; RT: retrotranscription*

| **Sample** | **RT enzyme** | **DNase treatment** |  |
| --- | --- | --- | --- |
| 1 | - | + | no cDNA, no EV-DNA |
| 2 | + | - | cDNA + EV-DNA |
| 3 | - | - | only EV-DNA |
| 4 | + | + | only cDNA |

| **Transcript** | **Sample 1**  no cDNA  no EV-DNA | **Sample 2**  EV-DNA  cDNA | **Sample 3**  only EV-DNA | **Sample 4**  only cDNA |
| --- | --- | --- | --- | --- |
|  |  |  |  |  |
| **Non-intron Spanning** |  |  |  |  |
| CASP8 | 0 | 3229 | 1855 | 0 |
| TNFRSF8 | 0 | 2200 | 1511 | 0 |
| B2M | 0 | 7622 | 1441 | 146 |
|  |  |  |  |  |
|  |  |  |  |  |
| **Intron Spanning** |  |  |  |  |
| PRKCD | 0 | 44 | 0 | 0 |
| TNFRSF10C | 0 | 31 | 0 | 0 |
| FCER1G | 0 | 995 | 0 | 10 |
|  |  |  |  |  |

**Supplementary Table S5.** Primary and secondary antibodies used for the immunoblotting experiment.

| **Antibody** | **Dilution** | **Company and catalog** |
| --- | --- | --- |
| Mouse anti-Flotillin-1 | 1:200 | BD Biosciences (610821) |
| Mouse anti-CD63 | 1:200 | Cell Marque (263M-15) |
| Mouse anti-Calnexin | 1:500 | Santa Cruz Biotechnology (AF18:sc-23954) |
| HRP-linked goat anti-mouse (from sheep) | 1:5000 | GE Healthcare Life Sciences (NA931-1ML) |
